# Local scattering ultrasound imaging

**DOI:** 10.1038/s41598-020-79617-z

**Published:** 2021-01-13

**Authors:** Alexander Velichko, Eduardo Lopez Villaverde, Anthony J. Croxford

**Affiliations:** Department of Mechanical Engineering, University of Bristol, Bristol, BS8 1TR UK

**Keywords:** Ultrasound, Acoustics, Imaging techniques, Mechanical engineering, Imaging techniques, Characterization and analytical techniques

## Abstract

Ultrasonic imaging is a widely used tool for detection, localisation and characterisation of material inhomogeneities with important applications in many fields. This task is particularly challenging when imaging in a complex medium, where the ultrasonic wave is scattered by the material microstructure, preventing detection and characterisation of weak targets. Fundamentally, the maximum information that can be experimentally obtained from each material region consists of a set of reflected signals for different incident waves. However, these data are not directly accessible from the raw measurements, which represent a superposition of reflections from all scatterers in the medium. Here we show, that a complete set of transmitter–receiver data encodes sufficient information in order to achieve full spatio–temporal separation of transmitter–receiver data, corresponding to different local scattering areas. We show that access to the local scattering data can provide valuable benefits for many applications. More importantly, this technique enables fundamentally new approaches, exploiting the angular distribution of the scattering amplitude and phase of each local scattering region. Here we demonstrate how the local scattering directivity can be used to build the local scattering image, releasing the full potential and richness of the transmit–receive data. As a proof of concept, we demonstrate the detection of small inclusions in various highly scattering materials using numerical and experimental examples. The described principles are very general and can be applied to any research field where the phased array technology is employed.

## Introduction

Ultrasonic imaging and the associated detection inside a random scattering medium is an indispensable tool in many practical applications across a broad range of fields, for example, in utero monitoring or weld inspection. However, this is a fundamentally challenging task because of coherent scattering noise, which is induced by the material’s microstructure and small material changes on the ultrasonic propagation path, resulting in low contrast. Recent advances in ultrasonic imaging have been based on the use of phased arrays. The ability to control the phase of each array element makes it possible to physically focus the incident wave at an arbitrary location inside the material. Alternatively, the transmit–receive array data can be collected using unfocused excitations, and then an image can be formed in post-processing by applying an imaging algorithm to the measured data. Two most common data acquisition schemes correspond to firing each array element individually^1,2^ (this process is also known as the Full Matrix Capture (FMC) in the field of non-destructive testing), or to firing all array elements with appropriate time-delays in order to generate a plane incident wave^3–5^ (plane wave imaging, PWI).

**Figure 1:**
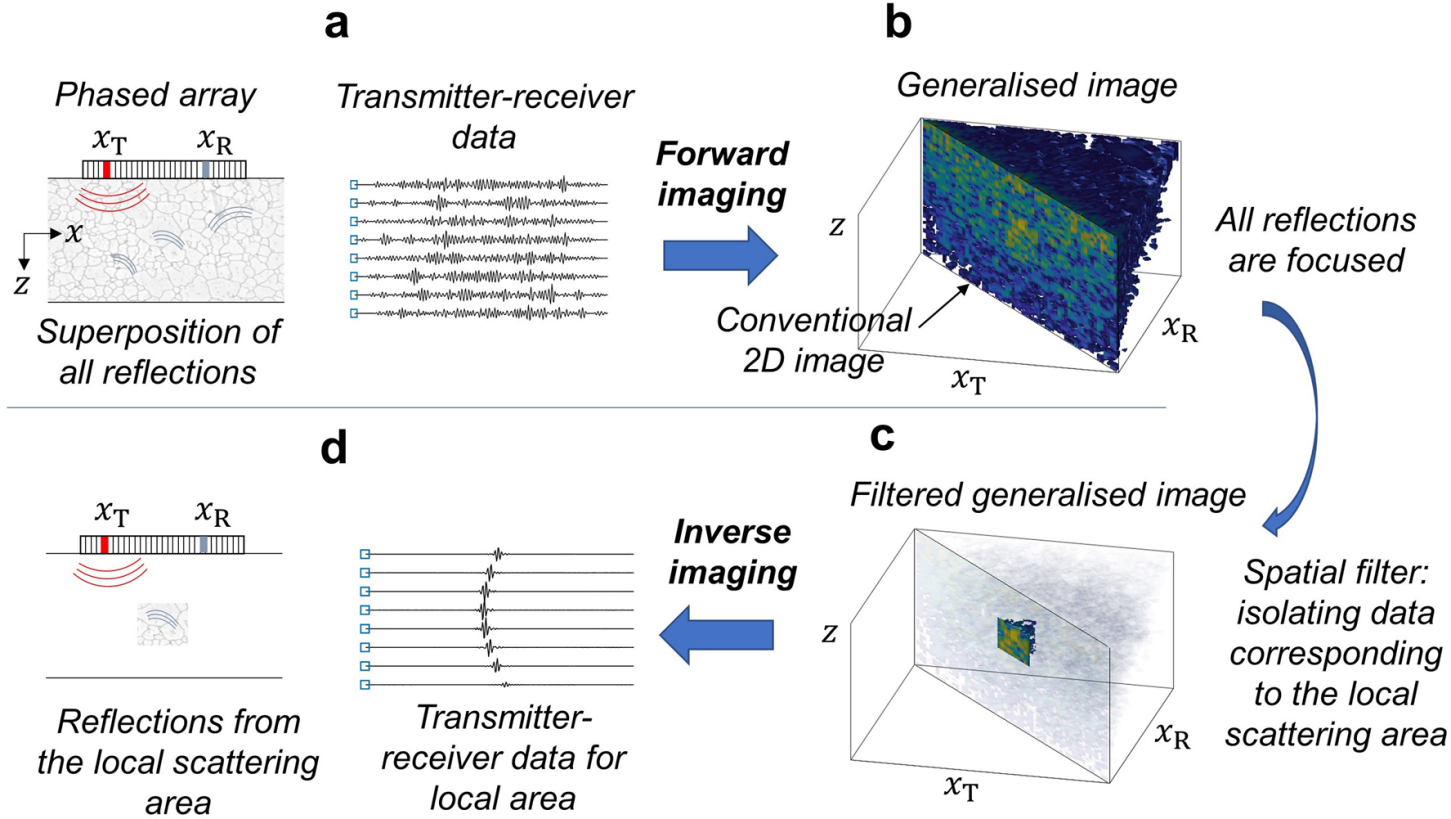
Reversible imaging concept. (**a**) Transmit–receive array data are collected by firing each array element and using all elements on reception. The data represents superposition of reflections from multiple scatterers in the material. (**b**) Transmit–receive data is converted into the generalised image with independent transmit and receive focusing points $x_T$ and $x_R$. The data corresponding to each scattering area is localised in the vicinity of the main diagonal $x_T=x_R$. (**c**) A spatial filter is applied to the generalised image in order to isolate responses from the local scattering area. (**d**) The filtered generalised image is then converted back into array data, providing isolated transmit–receive signals for the local scattering area.

The ultimate goal of imaging is to obtain the maximum possible information about each local region inside the medium. This includes detection, localisation and characterisation (for example, estimation of the size and material properties of the local feature). Recently, several methods based on the full transmitter–receiver data have been proposed, aiming to improve imaging and characterisation of scattering materials^6–21^. However, these techniques make use of only some specific properties of the measured response and do not exploit the full information contained in the transmitter–receiver dataset. On the other hand, multi-element array measurements essentially represent the transmitter–receiver datasets corresponding to each local scattering area. Importantly, the local array data contains information about the angular distribution of the scattering amplitude and phase. Access to these local scattering characteristics could provide significant benefits for many applications in a diverse range of fields, including detection of weak targets and material characterisation^14–17,22^. However, in a scattering material the transmitter–receiver response at each time instance contains a superposition of signals from all scatterers in the material, so the local transmit–receive dataset is not directly available from the raw array data (Fig. 1a).

Spatial selectivity can be achieved by performing dynamic focusing in transmission and reception, which transforms the measured array data into a reconstruction image where the scatterers can be spatially localised. Among many different imaging methods, delay-and-sum (DAS) is the most common approach being used for medical imaging and non-destructive testing applications, because of its low complexity, robustness and real-time imaging capability. The image value at each pixel is given by the summation of all transmitter–receiver signals with appropriate time-delays, and the image amplitude provides an estimation of the average reflectivity of the scatterers. However, the information about phase and amplitude distribution for each local scattering area is lost in this irreversible image formation process. In other words, it is generally impossible to convert a conventional image (for example, 2D delay-and-sum image obtained using 1D linear phased array) back into transmitter–receiver array data without making some specific assumptions about the scattering mechanism. For example, a common approach is to consider each image pixel as an omni-directional point scatterer with the reflectivity equal to the image amplitude.

The reversible imaging concept^23^ allows this problem to be overcome and is illustrated in Fig. 1. Firstly, the complete transmit–receive array data is mapped from the raw $(x_T,x_R,t)$ domain (here $x_T, x_R$ are positions of transmitter and receiver array elements and *t* is time) into the imaging domain, $(x_T,x_R,z)$, with independent transmit and receive focusing points $x_T, x_R$ at each depth *z* (Fig. 1b). The obtained image is referred to as a generalised image, because it contains much more information, than is available from the conventional reflectivity image. Indeed, the conventional image corresponds to the main diagonal of the generalised image, $x_T=x_R$. However, the off-diagonal part is crucial for the inverse imaging operation. Moreover, by using the off-diagonal data it is possible to estimate the multiple-scattering rate^24,25^, and the focusing quality of the image^25^. In this paper we use the back-propagation of angular spectrum imaging algorithm^23,26^, where all data processing steps are performed using Fourier transforms and, therefore, are fully reversible. Note, that the back-propagation and the inverse imaging methods can also be represented in the delay-and-sum form with some weighting coefficients^27^ (also see Supplementary Information). In the second step, the generalised image area corresponding to the local region-of-interest is spatially filtered from the whole image and converted back into the raw array data domain by applying inverse imaging operations (Fig. 1c). The resulting local array data corresponds to the geometry where only one local scattering area is present in the otherwise homogeneous material, and, therefore, provides full spatio-temporal separation of responses from different scatterers in the medium (Fig. 1d).

The described principle is very general and can be applied to any field where phased array technology is used or available. One immediate benefit is the possibility of using existing signal processing methods, which were developed under the assumption of a limited number of scatterers inside the observed medium. For example, it was shown that localisation of scatterers with resolution better than the classical diffraction limit (super-resolution) is achievable using multiple signal classification (MUSIC) and decomposition of the time-reversal operator (DORT) techniques^28,29^, but only when the number of scatterers is smaller than the number of array elements. However, these methods become less effective or fail if applied to the complete transmit–receive data, corresponding to the whole imaging region.

More importantly, access to the local transmit–receive data makes it possible to apply fundamentally new imaging approaches, one of which is the main subject of this paper. The extracted local array data can be processed further in order to obtain specific properties of the local scattering region. In this paper, the local array data is converted into the far-field scattering amplitude (see Supplementary Fig. 1), which is also called the scattering matrix, and has been extensively used for the characterisation of small (order of wavelength) scatterers^22,30–32^. In general, the scattering amplitude is a complex valued function of incident angle, scattered angle and frequency. The advantage of this step is that effects related to the measurement system, such as array element directivity and geometrical beam-spreading, are separated from the local scattering amplitude. Therefore, contrary to the image or the local array data, the scattering matrix represents the fundamental characteristic of the local scattering area alone and does not depend on the parameters of the array. As such this represents the fullest description of a material region and this paper explores how the extra information may be leveraged.

The scattering amplitude extraction can be performed for every location in the material, complementing the delay-and-sum image. It fully exploits the richness of the transmit–receive array data allowing access to information about the local physical properties beyond that available from conventional images. Here we show how to use this information to build a local scattering image and to improve detectability of weak inclusions. Both simulations and experimental results verify the superior performance of the proposed method and demonstrate its power over conventional image based detection approaches.

This paper is organized as follows. In the “Methods” section the general principle of the proposed local scattering imaging method is introduced and illustrated on the simulated example of a weak inclusion in highly scattering material. Then the performance of the method is evaluated on experimental data obtained from fabricated scattering samples with various imbedded inclusions in the “Results” section. The limitations and important implementation details of the local scattering method are considered in the “Discussion” section. The paper is complemented by the Supplementary information, which contains detailed mathematical formulations of the scattering matrix extraction algorithm and applied statistical data analysis.

## Methods

### Imaging algorithm and scattering matrix extraction

**Figure 2:**
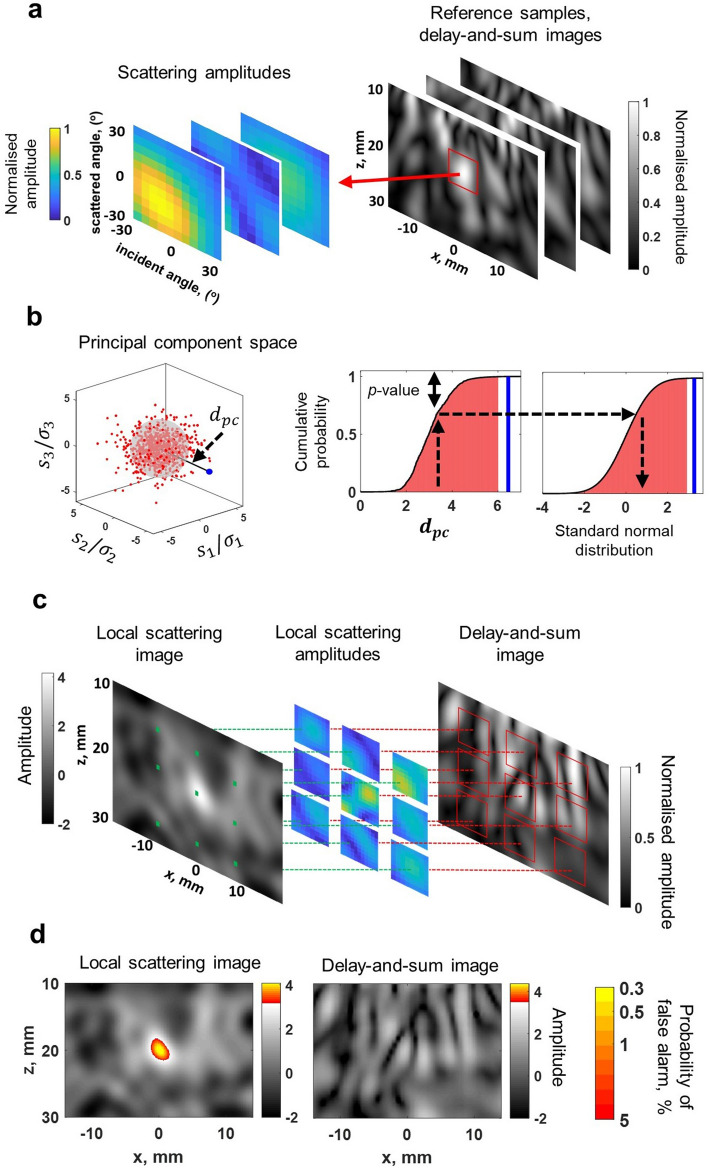
Local scattering imaging: principle and numerical example. (**a**) Local transmitter–receiver data is extracted from every location of reference samples and converted into local scattering matrices. Only absolute values of complex scattering matrices are shown. (**b**) Scattering matrices are converted into the principal component space and its distribution is characterised by the normalised distance $d_{pc}$. The gray sphere in the principal component space corresponds to the value of 3 standard deviations, $s_n/\sigma _n=3$. The cumulative distribution function is estimated, and *p*-value is assigned to each scattering matrix. Finally, *p*-values are mapped into the standard normal distribution. Blue markers correspond to the scattering matrix, extracted from the test sample with the inclusion. (**c**) Local scattering amplitudes are extracted from every location of the test sample (shown by the green dots), converted into the local principal component space and then into the standard normal distribution (*z*-scores) using mapping shown in Fig.2b. The resulting local scattering image represents a Gaussian random field. (**d**) The same principle can be applied to any imaging parameter, including delay-and-sum image amplitude. This allows the display of different images (delay-and-sum and local scattering image) on the same scale and defined global false alarm rate (shown as the second colorbar).

The fundamental concept of the proposed imaging method is to use the vast amount of information encoded in the scattering matrix for detection of weak inhomogeneities inside scattering media. In this context our goal is to obtain a quantitative measure of probability that the scattering behaviour of the local area can be explained by the background material microstructure. The approach is illustrated using simulated data in Fig. 2. A finite-element (FE) 2D model was implemented in the Pogo software package^33^. The material properties were chosen close to those of copper with the longitudinal sound speed of 4690 m/s and a mean grain size of $100~\mu \hbox {m}$. These values were selected due to the highly scattering nature of such a material, enabling exploration of the basic approach. The backscattered signals in this case are generated by ultrasonic scattering at the grain boundaries. The full matrix capture data were generated for a 2.5 MHz 64-element linear array (0.5 mm pitch) in the direct contact configuration (as shown in Supplementary Fig. 1a) and the imaging was performed in the area $(x,z) \in [-15,15]\times [10,30]~\hbox {mm}$ directly below the array, where the centre of the array is at the origin.

The forward and inverse imaging operators were based on the back and forward propagation of the angular spectrum of transmit–receive array data. One efficient numerical implementation was to compute images in the Fourier domain using the fast Fourier transform algorithm^23,34,35^. Alternatively, we used the equivalent delay-and-sum representation (see Supplementary Information). This approach was implemented on a graphics processing unit (GPU) with the CUDA programming model and run on MATLAB (The MathWorks Inc., Massachusetts, USA). The computer was a standard desktop with Intel Core i7-6700 CPU @3.4GHz (16 GB RAM) and an NVIDIA GPU GeForce GTX 1060 (6 GB RAM). This implementation enabled a high extraction rate of 30 local scattering amplitudes per second for a 64-element array, significantly reducing the computation time.

A 2 mm diameter inclusion (with the same elastic constants as the background material, but increased density, so the longitudinal velocity decreased by 21% relative to the velocity in the background material) was placed at a depth of 20 mm directly below the array centre. Scattering matrices, $S(\mathbf{r})$, were extracted at 1 MHz from each location, $\mathbf{r}$, of the image with 2 mm steps using a $7~\hbox {mm} \times 7~\hbox {mm} \times 7~\hbox {mm}$ cubic window (7 mm = 1.5 wavelengths at 1 MHz) in the generalised image domain (Fig. 2a). The choice of frequency and spatial sampling window size is very important and is based on the requirement to operate predominantly in the single scattering regime (see “Discussion” and Supplementary Information for more details). In all cases, we used real and imaginary parts of the extracted complex valued scattering amplitude, therefore taking into account both its magnitude and phase. Finally, the scattering amplitudes were normalised by the average image speckle intensity at each depth^36^, compensating for the effect of material attenuation (see Supplementary Information and Supplementary Fig. 2). Technical details of the scattering matrix extraction method are given in Supplementary information (Supplementary sections 1, 2, 3 and 4).

### Statistical analysis

A probability value, $p(S(\mathbf{r})|H_0)$, was assigned according to how likely the measured scattering matrix $S(\mathbf{r})$ is to occur under the null hypothesis, $H_0$, that there are no inclusions in the material distinct from the random variations of the background microstructure. This is the *p*-value of the scattering matrix, which can be expressed as $p(\mathbf{r})=1-P_{cdf}(\mathbf{r}, S)$ for the one-tailed test, where $P_{cdf}(\mathbf{r},S)$ is the cumulative distribution function of background scattering matrices at the location $\mathbf{r}$ (Fig. 2b). In order to estimate the probability distribution $P_{cdf}(\mathbf{r},S)$, array datasets for 250 different grain structure realisations without inclusions were simulated. The statistical distribution of scattering matrices is convenient to study in the principal component space, which is defined by applying principal component analysis to the database of the background scattering matrices^22^.

The normalised distance, $d_{pc}$, in the principal component space was defined as

1 $$\begin{aligned} d_{pc}(S)=\sqrt{\sum \limits _{n=1}^N \left( \frac{s_n}{\sigma _n} \right) ^2} \end{aligned}$$

and was used as the statistical parameter of the distribution (see Fig. 2b). In this expression $s_n$ and $\sigma _n$ are the *n*-th principal component coefficient of the scattering matrix *S* and its standard deviation, respectively. The number of principal components used, *N*, was determined according to the condition $\sigma _n/\max \limits _n(\sigma _n) \le 0.1$.

The cumulative distribution function, $P_{cdf}(\mathbf{r},d_{pc})$, was estimated at each location from the reference dataset, and *p*-images $p(\mathbf{r})$ were calculated. Note, that the $P_{cdf}$ was determined experimentally up to an amplitude threshold, corresponding to $P_{cdf} = 0.9$. The tail of the distribution, which is the most important for the probability of false alarms analysis, was approximated by fitting the Weibull distribution function to the top $10\%$ of samples in the database^37^.

### Global false alarm rate

The detection criterion is based on the threshold (significance) level for the *p*-value. Note, that $p(\mathbf{r})$ gives the local probability of false alarms (the false positives rate) at the point $\mathbf{r}$. However, the detection is performed on the whole image, so it is more appropriate to use a global threshold (global probability of false alarms) for the entire image. The global false alarm rate represents a probability that at least one pixel in the image exceeds a given threshold. This is the problem of multiple comparisons. The difficulty in this case lies in the fact, that neighbouring pixels in the image are not independent due to the finite size of the point spread function, so the conventional Bonferroni correction is too conservative. One alternative is to experimentally estimate the global false alarm rate as a function of threshold from the reference array data. However, in practice this is not always possible, especially for small false alarm probabilities, because of the relatively small number of reference samples. Instead, *p*-values were mapped into the standard normal distribution by the following operator, giving the *z*-score image $z_S(\mathbf{r})$ (see Fig. 2b):

2 $$\begin{aligned} z_S(\mathbf{r})=P^{-1}_{cdf,N(0,1)}\left( P_{cdf}(\mathbf{r},d_{pc})\right) , \end{aligned}$$

where $P_{cdf,N(0,1)} (x) = 0.5 \left[ 1+\text {erf}( x/\sqrt{2} ) \right]$ is the cumulative distribution function of the standard normal distribution, and $\text {erf}(x)$ is the error function.

The main benefit of this transformation is that the image $z_S(\mathbf{r})$ represents the Gaussian random field. For the Gaussian random field an analytical expression for the global probability of false alarms, $p_z(z_S(\mathbf{r}))$, was derived by Worsley *et* *al*.^38^, which parametrically depends on the number of the resolution elements, or resels, in the imaging area. The resel size can be approximated as the full-width at half-maximum of the point spread function^38^. Alternatively, we estimated the false alarm rate using images obtained from all reference datasets. However, this approach could not be used to reliably define the threshold for low probabilities of false calls because of the small number of reference images. We then determined the resel size by fitting the analytical expression to the estimated probability of false alarm curve (Supplementary Fig. 4 and Supplementary Table 1). After that, the threshold for any value of the global probability of false alarm $p_{pfa}$ was calculated as the inverse mapping $z_S = p_z^{-1}(p_{pfa})$ (see Supplementary Fig. 4a).

### Local scattering image

The image obtained using the described procedure provides quantitative information about local scattering properties of the material, and, therefore, is referred to as a local scattering image, or S-image (Fig. 2c). Note that the *z*-score can also be interpreted as the Contrast-to-Noise ratio (CNR). Indeed, the Contrast-to-Noise ratio can be defined as a ratio of the difference between the signal and average noise and the standard deviation of the noise. For the standard normal distribution the mean is zero and the standard deviation is one, so the Contrast-to-Noise ratio in this case equals the signal, or the *z*-score.

The described approach is very flexible and can be applied to any imaging parameter, including the image amplitude. This allows definition of the global false alarm rate for delay-and-sum images, and display of delay-and-sum and local scattering images on the same scale, providing a direct quantitative comparison of the newly developed approach to current best practise. Fig. 2d shows the resulting delay-and-sum and local scattering images for the considered case of the inclusion in grainy material. It can be seen that the inclusion is totally undetectable in the conventional image. On the contrary, the local scattering image provides enough sensitivity to clearly detect the inclusion. The superior performance of the local scattering method is further highlighted by the peak image value of 4.1, which corresponds to a global false alarm rate of $0.3\%$. In contrast, the peak value of the normalised delay-and-sum image is 2.3, which corresponds to a global false alarm rate of nearly $100\%$ (Supplementary Fig. 4a). Therefore, the improvement in the local scattering image in this case is more than two orders of magnitude in terms of the false alarm rate.

In order to study statistical performance of the local scattering imaging method, 10 different inclusion types with various sound speeds and 100 different realisations of grain structure for each inclusion type were modelled. The results are presented in Supplementary Fig. 5.

## Results

For the experimental measurements we fabricated samples using two-component (base plus curative) mold rubber systems (Smooth-on, Inc., Pennsylvania, USA). As the first experimental demonstration we performed measurements on a cast silicone-rubber sample (Ecoflex 00-50 series, cure time 3 hours) of $90~\hbox {mm}~\times 90~\hbox {mm} \times 30~\hbox {mm}$ size, uniformly mixed with fine sand (0.5 mm average grain size). In mixing sand into the component it was possible to control the level of scattering to recreate noisy materials, while being able to reliably embed inclusions (something historically challenging with engineering parts). The density and the sound velocity of the solidified rubber were $1.3~{\hbox {g/cm}}^3$ and 1025 m/s, respectively. The density of the sand in the fabricated samples was $10^{-2}~{\hbox {g/cm}}^3$, which corresponded to 1 g of sand per 100 ml of the liquid silicone-rubber solution. The sample contained two rows of 4 inclusions, which were fixed on steel pins of 0.55 mm diameter at 10, 15, 20 and 25 mm depths. All inclusions were 3 mm diameter and were cut from the same material, but with 3 times higher sand density, resulting in different scattering at the inclusions. The full dataset of transmitter–receiver signals was collected using a 64 element 2.5 MHz linear array with 0.5 mm pitch, 0.15 mm inter element spacing, 15 mm element elevation (Imasonic, Besancon, France) and 10 MHz sampling frequency. All measurements were performed in a direct contact configuration with ultrasonic gel as a couplant.

Reference data were collected by scanning the array over the part of the sample without inclusions. The array was scanned along the sample, then rotated by $90^{\circ }$ and scanned again with a 5 mm step. In total 75 reference datasets were acquired.

The local scattering data were extracted from the imaging area $-15~\hbox {mm}~\le x \le ~15~\hbox {mm}$, $5~\hbox {mm}~\le z \le ~25~\hbox {mm}$ with a 0.5 mm step. The local scattering images were then interpolated into a finer grid with 0.1 mm step using sinc interpolation. An angular filter of $30^{\circ }$ was applied to the array data and the scattering amplitudes were extracted in the angular range of $\pm 30^{\circ }$. This filtering helped to suppress noise and aliasing artifacts related to the array elements undersampling (with the arrays not being designed for this material so being incorrectly sampled) but had relatively small effect on the image resolution^39^.

**Figure 3:**
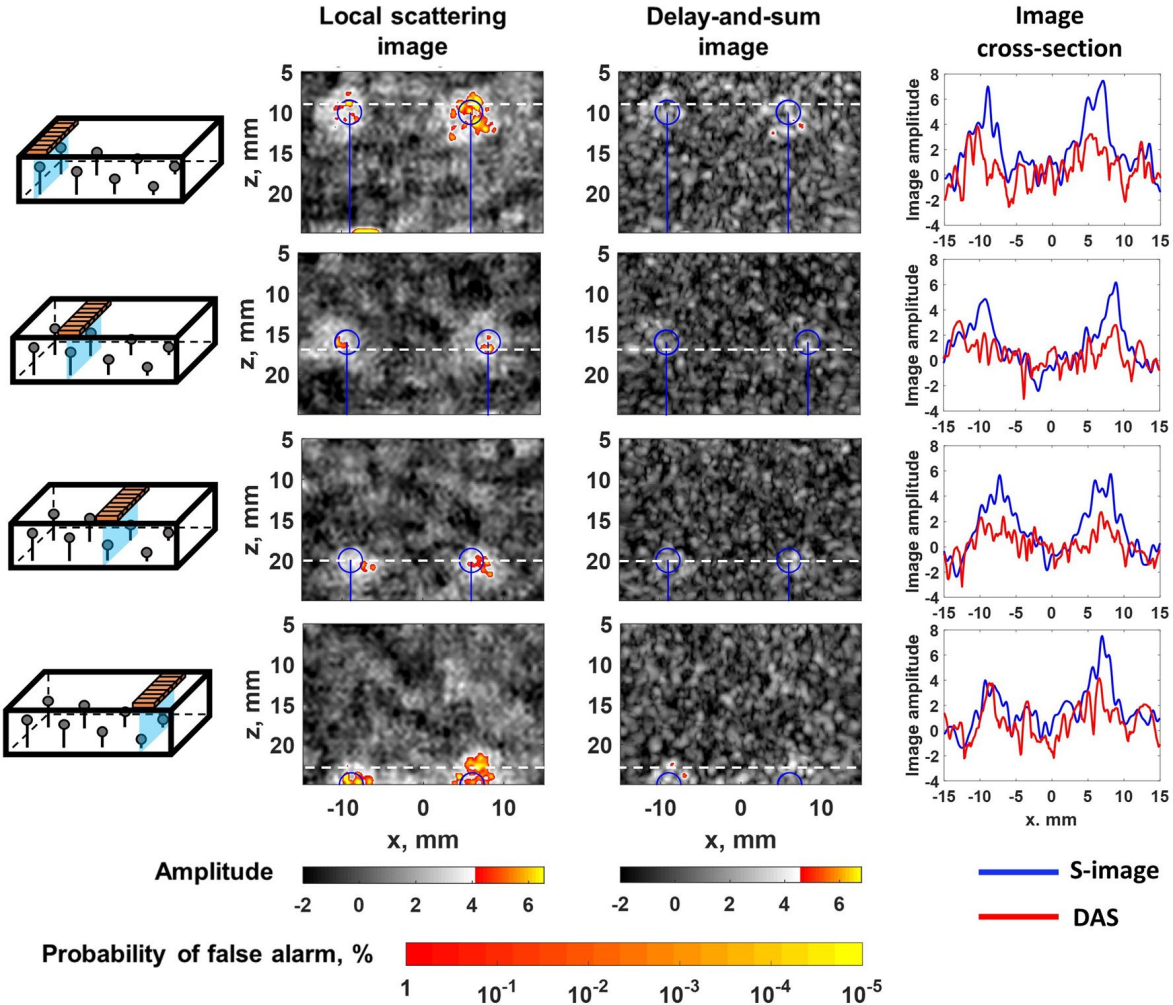
Experimental example of local scattering imaging: silicone-rubber sample. Examples of local scattering and delay-and-sum images of inclusions located at different depths inside the silicone-rubber sample. Blue circles indicate the nominal position of inclusions. The global false alarm rate is shown as the second colorbar. Image cross-sections correspond to depth positions indicated by white dashed lines.

**Figure 4:**
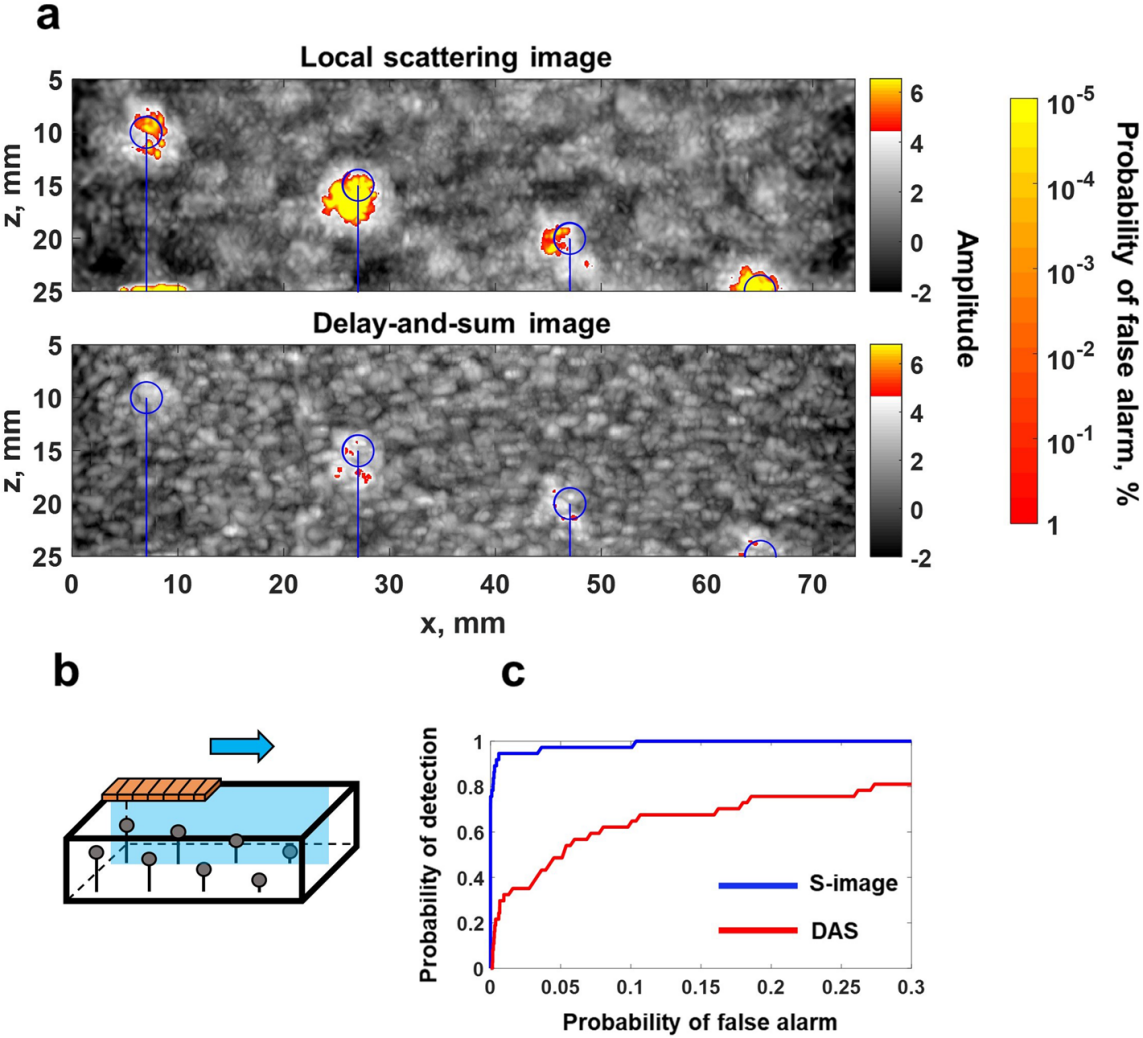
Experimental example of local scattering imaging: scanning along the silicone-rubber sample. (**a**) Local scattering and delay-and-sum images, the value of each pixel equals to the maximum over all scanning array positions. Blue circles indicate the nominal position of inclusions. The global false alarm rate is shown as the second colorbar. (**b**) Diagram illustrating the geometry of the sample. (**c**) Receiver operating characteristic (ROC) curve, summarising the detection statistics over all scanning positions for all inclusions.

A Gaussian frequency filter with center frequencies of $f=1.2, 1.5, 1.8~\hbox {MHz}$ and a 100% fractional bandwidth was applied to the measured data, and local scattering and delay-and-sum images were constructed using the procedure described in the previous section, and illustrated in Fig. 2, at each frequency. Then the images at different frequencies were fused together by taking the maximum image amplitude (*z*-score) at each pixel, equivalent to taking the minimum *p*-value. Fused images were again converted into *z*-scores by evaluating the cumulative distribution function, and the global probability of false alarm as a function of the amplitude threshold was determined in each case (see Supplementary Fig. 4b). The results of this experiment are summarised in Figs. 3 and 4. Figure 3 presents images with the array positioned directly above inclusions. It clearly shows, that delay-and-sum images mostly fail to detect inclusions, in contrast to local scattering images, which provide sensitivity 1-2 orders of magnitude higher in terms of the probability of false calls. The array was also scanned above one row of inclusions with a 2 mm step as illustrated in Fig. 4b (see Supplementary video 1). Figure 4a shows the combined image from this scan, where the value of each pixel is equal to the maximum over all scanning positions. The detection rate was evaluated using the receiver operating characteristic (ROC) curve, which displays the probability of detection as a function of probability of false alarms. Figure 4c shows ROC curves, which give information about the statistical performance of the imaging methods over all scanning positions. In this case each inclusion was considered as detected if the image amplitude was above the threshold in the area $5~\hbox {mm}~\times ~5~\hbox {mm}$ around the nominal center of the inclusion (taking into account possible inaccuracy of the nominal location and size of inclusions). Note, that the probability of false alarms corresponding to each threshold value was calculated using a theoretical expression (see Supplementary section 6). These results demonstrate, that the local scattering image allowed detection of inclusions at almost all scanning positions, in contrast to the delay-and-sum method, where detection was possible at only a small number of specific array locations relative to inclusions.

The second set of experimental measurements were performed on a similar specimen, but fabricated using urethane-rubber (Vytaflex 50 series, cure time 16 hours). The material density in this case was $2~{\hbox {g/cm}}^3$, the sound speed was 1500 m/s and the sand density was the same as in the previous case. The size of the sample was $143~\hbox {mm}~ \times ~35~\hbox {mm}~ \times ~30~\hbox {mm}$. Inclusions of the same type as for the silicone rubber sample (inclusions were cut from the silicone-rubber sample as in the previous example, so in this case they differed from the background material by the sand concentration as well as material properties) were placed at 15, 20 and 25 mm depths. In order to collect reference data the array was scanned along the part of the sample without inclusions with a 2.5 mm step, resulting in 38 datasets.

**Figure 5:**
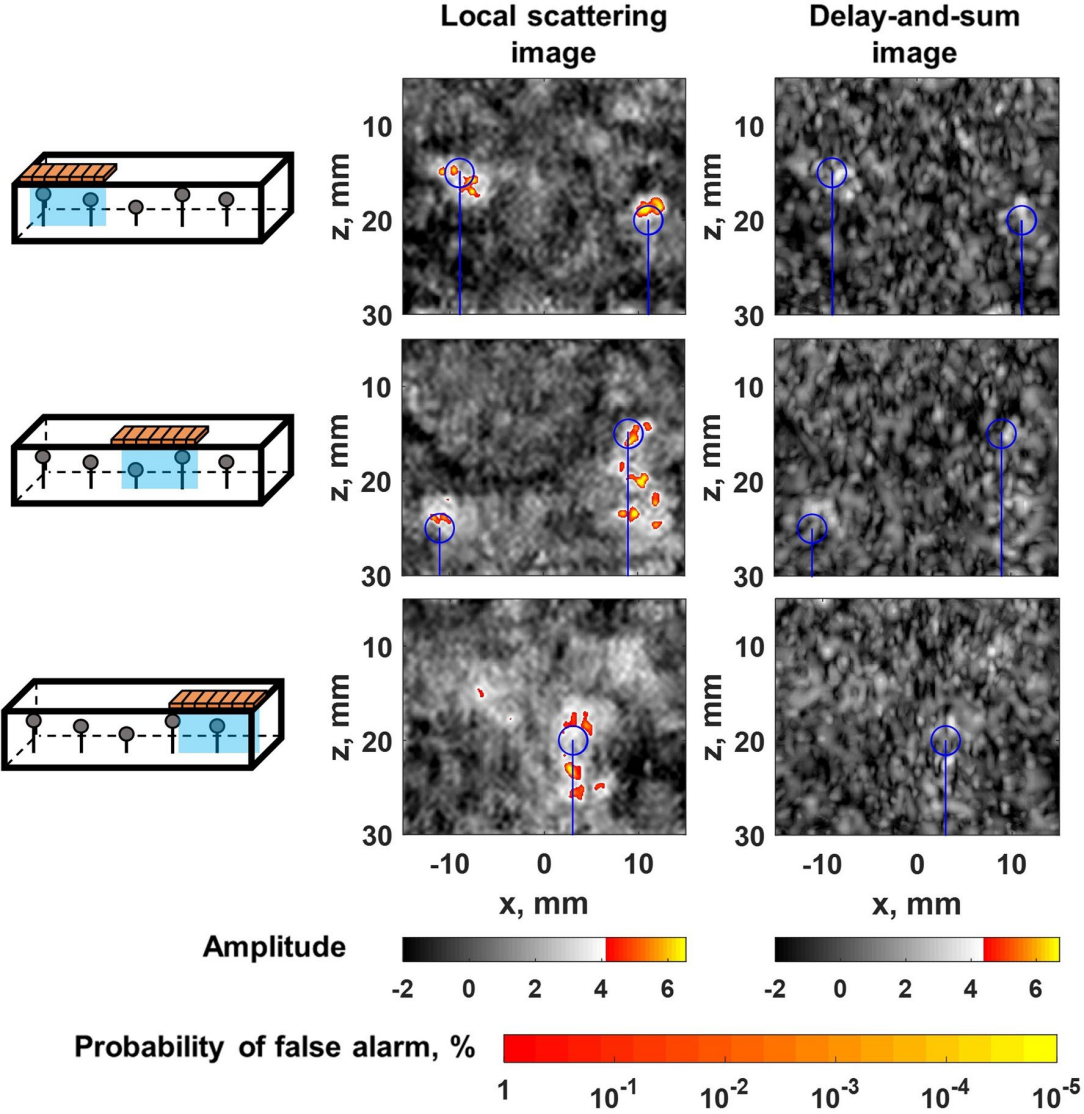
Experimental example of local scattering imaging: urethane-rubber sample. Examples of local scattering and delay-and-sum images of inclusions located at different depths inside the urethane-rubber sample. Blue circles indicate the nominal position of inclusions. The global false alarm rate is shown as the second colorbar.

**Figure 6:**
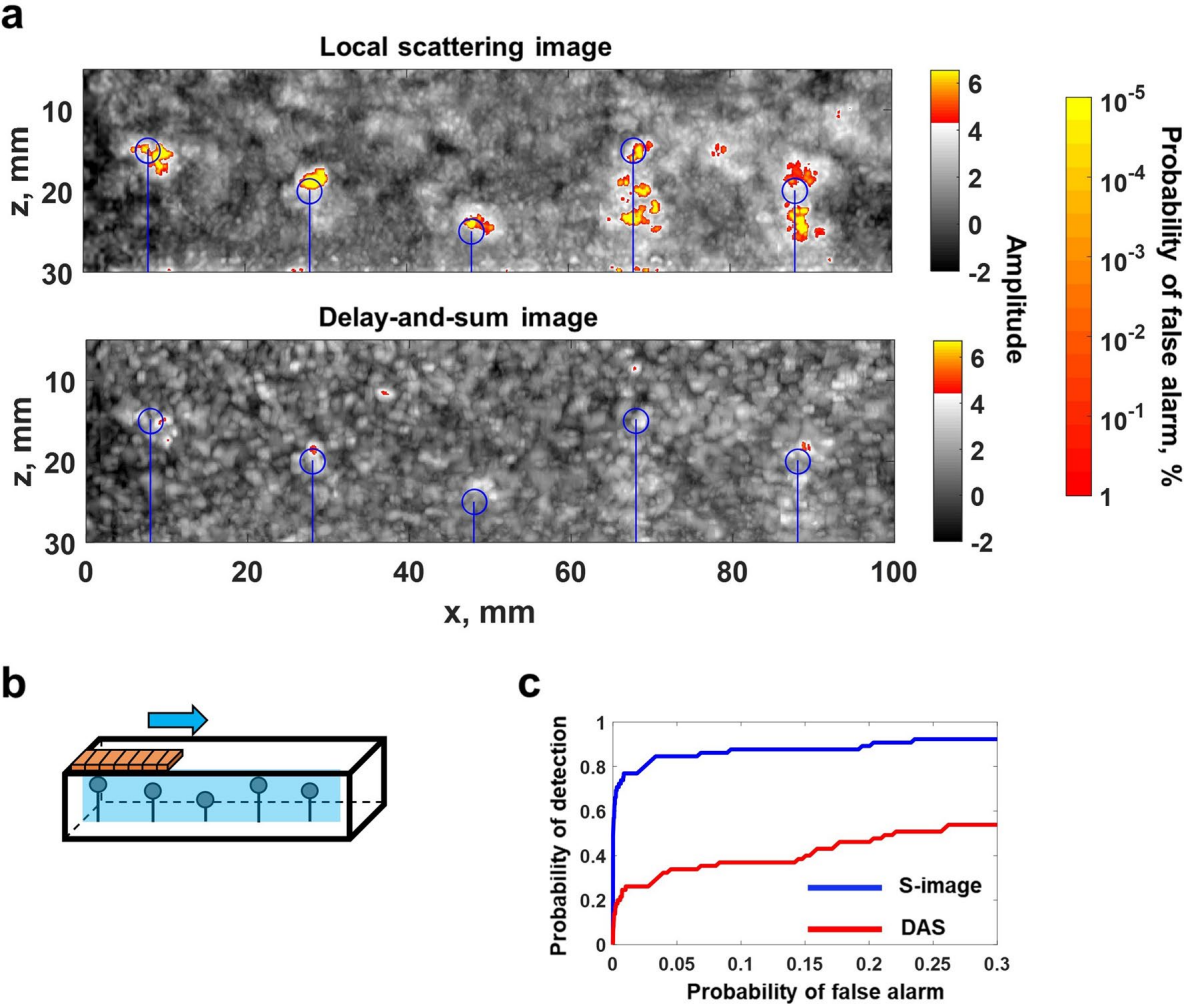
Experimental example of local scattering imaging: scanning along the urethane-rubber sample. (**a**) Local scattering and delay-and-sum images, the value of each pixel equals to the maximum over all scanning array positions. Blue circles indicate the nominal position of inclusions. The global false alarm rate is shown as the second colorbar. (**b**) Diagram illustrating the geometry of the sample. (**c**) Receiver operating characteristic (ROC) curve, summarising the detection statistics over all scanning positions for all inclusions.

The local scattering data were extracted from the imaging area $-15~\hbox {mm}~\le x \le ~15~\hbox {mm}$, $5~\hbox {mm}~\le z \le ~30~\hbox {mm}$ with 0.5 mm step. The imaging was performed at 1.2, 1.5 and 2 MHz and final images were obtained by fusing the images at different frequencies. The array was scanned over inclusions with a 2 mm step (Fig. 6b, Supplementary video 2) and examples of the local scattering and delay-and-sum images are presented in Fig. 5. Figure 6a shows the images combined for all array positions with the maximum image amplitude value at each location, complemented by ROC curves in Fig. 6c. The significant enhancement in sensitivity, given by the local scattering approach is clearly demonstrated. Interestingly, in some cases, the local scattering image is sensitive enough to show indications of the steel pin, which supports the inclusion, and also the fixing clay layer at the bottom of the images, used to fix the pin to the bottom of the moulding container.

## Discussion

The local ultrasonic scattering data encode significant information about material properties. This provides the basis for the proposed method, which extracts the local scattering data from the array measurements and then uses the angular distribution of the scattering amplitude and phase to greatly enhance the sensitivity of the image. It is well known that the physical nature of scattered signals is determined by the size of microstructural inhomogeneities relative to the wavelength, and, as a result, strongly depends on the frequency of the incident wave. When the frequency increases, the multiple-scattering contribution in the image also increases, contaminating the extracted local scattering amplitudes. Therefore, it is crucial to choose the imaging frequency in the predominantly single-scattering regime. The proportion of the multiple scattering in the image can be directly obtained from the generalised image data^24,25^. Based on the simulations and experimental examples we estimated, that the local scattering method is applicable when the multiple scattering rate is approximately smaller than 25% of the total image intensity (see Supplementary Fig. 3). Note that for the experimental samples another limiting factor affecting the imaging frequency was array element under-sampling and high absorption at higher frequencies. The requirement for single scattering means we are likely operating in domains with good propagation performance allowing decent measurement range in difficult materials. It was shown that the reliability of ultrasonic images is directly related to the multiple scattering rate^24^, so the ability to estimate the appropriate measurement range in itself is a valuable factor in practical applications.

Another important imaging parameter is the size of the spatial filter in the generalised imaging domain for extraction of the local array data. This filter should be as small as possible in order to improve the resolution of the local scattering image, but at the same time large enough in order to contain all information about the local area. These requirements result in the size of the extraction window approximately equal to the size of the point spread function. For experimental examples considered in this paper we estimated that the optimal size of the spatial filter corresponds to 3$\lambda$, where $\lambda$ is the wavelength at the imaging frequency. Note, that the single scattering contribution is concentrated in the vicinity of the main diagonal of the generalised image, $x_T=x_R$, and off-diagonal values are more sensitive to the multiple scattering contribution^24^. Therefore, the spatial filter was additionally restricted in the off-diagonal region of the generalised image by the condition $|x_T-x_R| \le 2 \lambda$.

As mentioned, the scattering matrix represents the fundamental scattering characteristic of the local material area alone and, contrary to the image, does not depend on the parameters of the array. However, there are two main factors, related to the array geometry, which need to be considered. Firstly, the angular range of the extracted scattering matrix is determined by the array aperture. Secondly, the shape of the spatial filter in the generalised imaging domain might be affected by the array element pitch. In this paper it is implicitly assumed that the array element pitch satisfies the Nyquist sampling criterion^39^, ensuring the grating lobe level is negligible everywhere in the generalised image. On the other hand, in many fields, including medical ultrasonic imaging it is common to use under sampled arrays with $\lambda$-pitch. In this case grating lobes appear in the generalised imaging domain^40^, and the size of the spatial filter must be specifically chosen to suppress them.

The improvement in the sensitivity of the local scattering image comes at a price of reduced spatial resolution, as determined by the size of the extraction window. This is common to all statistical parametric imaging approaches, constructed using a sliding window, for example, Nakagami images^41^. The image resolution can be characterised by the size of the resolution element, or resel (see Methods and Supplementary Information). From the values, presented in Supplementary Table 1, it follows that the resolution of the local scattering images is approximately half that of conventional images. This represents a significant advance over other statistical parametric approaches. For example, for Nakagami imaging the typical window size, required to obtain enough independent image pixel values for the statistical analysis, is three times the pulse length of the incident wave^41^. On the contrary, in our method the window size was about one pulse length.

The final comment is related to the data acquisition scheme. In this paper the measured array dataset represented signals from all transmitter–receiver combinations. However, the method is applicable to any data collection strategy, where the reversible imaging operation is possible. For example, it has been recently shown that local scattering information can be extracted from plane wave data^40^. This opens up the possibility of combining the enhanced sensitivity of the local scattering imaging with the high frame rate using plane wave illuminations^3,4,15,16^.

## Conclusion

In this paper, we have introduced a new general approach for imaging in complex scattering medium. The key idea is to take advantage of all the information measured by an array of sensors about each local material area. This contrasts with most ultrasonic array imaging approaches which typically rely on averages of recorded information. The complete information represents a set of transmitter–receiver signals as if only one local scattering area was present in otherwise homogeneous material, and we have shown how it can be extracted from a complete measurement dataset. This is significant because access to the local scattering data provides information about the local physical properties beyond that available from conventional images and, therefore, can be highly beneficial for many applications. Most importantly, it allows fundamentally new methods to be developed for detection, localisation, and characterisation of targets in scattering materials or materials with low contrast. As a proof of concept, we have experimentally demonstrated one such approach to the detection of small inclusions in various highly scattering materials, which are invisible using conventional imaging techniques. The described methodology is very general and can be applied to any research field, where phased array technology is employed and may also potentially be expanded to other imaging modalities.

## Supplementary Information

Supplementary Information.

Supplementary video 1.

Supplementary video 2.

## Data Availability

The datasets generated during the current study are available from the corresponding author on reasonable request.
