# Supplementary material for: Local scattering ultrasound imaging
Source: Sci Rep. 2021 Jan 13;11:993. doi: 10.1038/s41598-020-79617-z (PMC7806797; doi:10.1038/s41598-020-79617-z)
Supplement: Supplementary file 1 — Supplementary Information. [file 41598_2020_79617_MOESM1_ESM.pdf]

Supplementary Information
Local Scattering Ultrasound Imaging

Alexander Velichko, Eduardo Lopez Villaverde, and Anthony J. Croxford
Department of Mechanical Engineering, University of Bristol, Bristol, UK

I. Reversible back-propagation imaging method

In this section the expression for the reversible back-propagation imaging operator is given. The array system geometry is schematically illustrated in Fig.1a.

Any time-domain signal $u(t)$ can be expressed as a linear superposition of its spectral components $u(\omega)$,

$$u(t) = \frac{1}{2\pi} \int u(\omega) e^{i\omega t} d\omega. \tag{1}$$

The back-propagation imaging method can be represented by a linear operator, B, which converts transmitter-receiver array data (Full Matrix Capture data), $g(t, x_T, x_R)$, into the generalised image, $b(z, x_T, x_R)$ [1],

$$b(z, x_T, x_R) = B[g(t, x_T, x_R)],\ B = F^{-1} H F. \tag{2}$$

Here F is a two-dimensional Fourier transform with respect to the array element coordinates x_T, x_R, F^{-1} is the inverse Fourier transform and H is the back-propagation of angular spectrum operator. Note that the physical meaning of the generalised image, $b(z, x_T, x_R)$, is transmitter-receiver array data, measured at time $t = 0$ by an array located at depth z [1]. Alternatively, it can be considered as beamforming with different transmit, (x_T, z), and receive, (x_R, z), focusing.

The Fourier transform operator F transforms the array data $g(t, x_T, x_R)$ into the angular spectrum $G(t, k_{x(T)}, k_{x(R)})$, where $k_{x(T)}$ and $k_{x(R)}$ are the wavenumbers in the x-direction for the transmitted and scattered waves, respectively:

$$G(t, k_{x(T)}, k_{x(R)}) \equiv F[g(t, x_T, x_R)] = \\ \int\int g(t, x_T, x_R)\, e^{i(k_{x(T)} x_T + k_{x(R)} x_R)}\, dx_T dx_R. \tag{3}$$

Note, that the back-propagation operation is based on the assumption that the transmitter and receiver elements are sensitive to the longitudinal wave mode only. Then it can be shown [1] that the angular spectrum, $G(t, k_{x(T)}, k_{x(R)})$, represents a 1D wave propagating in the z direction with the wavenumber $k_z = k_{z(T)} + k_{z(R)}$. Here $k_{z(T)} = \sqrt{k^2 - k^2_{x(T)}}$, $k_{z(R)} = \sqrt{k^2 - k^2_{x(R)}}$ are the wavenumbers in the z-direction for the transmitted and scattered wave, and $k = \omega/v$ is the scalar wavenumber, where v is the velocity of the longitudinal wave. The back-propagation of

the angular spectrum operator H converts the time data $G(t, k_{x(T)}, k_{x(R)})$ into a function of propagation distance, $h(z, k_{x(T)}, k_{x(R)})$, and can be written in the form:

$$h(z, k_{x(T)}, k_{x(R)}) \equiv H[G(t, k_{x(T)}, k_{x(R)})] = \frac{1}{2\pi} \int G(\omega, k_{x(T)}, k_{x(R)})\, e^{ik_z z}\, d\omega. \tag{4}$$

Note that for the spatial wavenumbers $k_{x(T)}, k_{x(R)} > k$ the spectrum $G(\omega, k_{x(T)}, k_{x(R)})$ corresponds to the exponentially decaying evanescent waves and it can be assumed that $G(\omega, k_{x(T)}, k_{x(R)}) = 0$ for $k_{x(T)}, k_{x(R)} > k$. It is convenient to change the integration variable from ω to k_z, then the integral (4) can be written in the form of a Fourier transform with respect to the variable k_z as

$$h(z, k_{x(T)}, k_{x(R)}) = \frac{1}{2\pi} \int G(\omega(k_z), k_{x(T)}, k_{x(R)}) \left(\frac{dk_z}{d\omega}\right)^{-1} e^{ik_z z}\, dk_z. \tag{5}$$

The conventional 2D image of scatterer position $I(x, z)$ is given by the pulse-echo data $x_T = x_R$ of the generalised image:

$$I(x, z) = b(z, x, x). \tag{6}$$

Therefore, the generalised image $b(z, x_T, x_R)$ contains more information than is necessary for localization of the scatterers. However, the extra information corresponding to the non-diagonal data $x_T \neq x_R$ is crucial for the inverse imaging.

Each operator in the expression (2) for the back-propagation operator B is reversible. Then the local array data, g_{local}, can be obtained by applying the inverse imaging operator to the spatially filtered generalised image:

$$g_{local}(t, x_T, x_R|x, z) = B^{-1}\left[b_{filt}(z, x_T, x_R|x, z) b(z, x_T, x_R)\right], \quad B^{-1} = F^{-1} H^{-1} F. \tag{7}$$

where B^{-1} is the inverse imaging operator, and b_{filt} is the filter in the generalised image domain for the region of interest around the location (x, z).

II. Asymptotic form of the back-propagation imaging method

In this section, expressions for the forward and inverse imaging operators in the delay-and-sum form are given. The derivations are based on the application of the stationary phase method to the integral representations of the imaging operators.

A. Forward imaging

The full derivation for the back-propagation operator is given in [2]. The resulting expression can be written as

$$b(z, x_T, x_R) = -\frac{1}{2\pi v} \int\int \frac{\cos\theta_T \cos\theta_R}{\sqrt{R_T R_R}} \frac{\partial}{\partial t} g\left(t = \frac{R_T + R_R}{v}, x'_T, x'_R\right) dx'_T dx'_R, \tag{8}$$

where $R_{T,R} = \sqrt{(x_{T,R} - x'_{T,R})^2 + z^2}$ and $\cos\theta_{T,R} = z/R_{T,R}$.

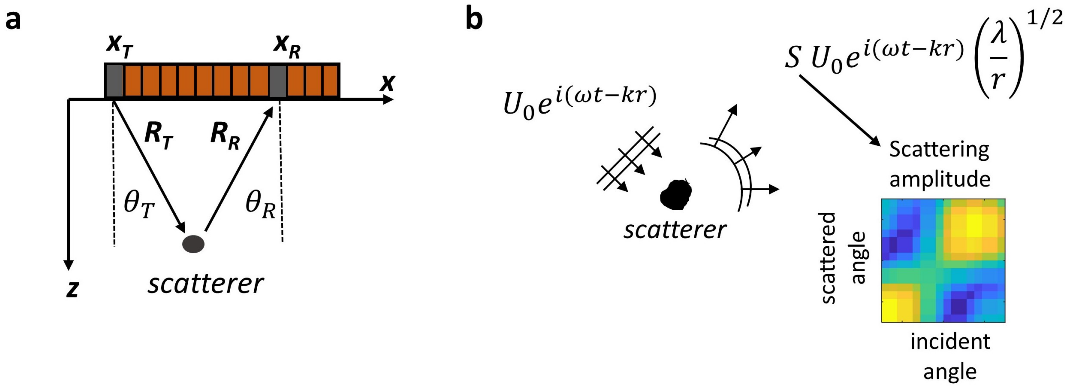

Fig. 1. (a) Array measurement geometry. (b) The far-field scattering amplitude S describes the directivity of the scattered wave when a scatterer is illuminated by an incident plane wave.

B. Inverse imaging

The frequency spectrum of the transmit-receive array data, $g(\omega, x_T, x_R)$, obtained using the inverse imaging operation from the generalised image $b(z, x_T, x_R)$ can be written as

$$g(\omega, x_T, x_R) = \int\int s_{inv}(z, x'_T, x'_R, \omega, x_T, x_R)\, b(z, x'_T, x'_R)\, dz dx'_T dx'_R, \tag{9}$$

where coefficients s_{inv} are given by

$$s_{inv} = \frac{1}{4\pi^2} \int\int \frac{dk_z}{d\omega} e^{-ik_{x(T)}(x_T - x'_T)}\, e^{-ik_{x(R)}(x_R - x'_R)}\, e^{-ik_z z}\, dk_{x(T)} dk_{x(R)}. \tag{10}$$

These integrals can be asymptotically evaluated as

$$s_{inv} = \hat{s}_{inv}\, i\omega e^{-ik(R_T + R_R)}, \quad \hat{s}_{inv} = \frac{1}{2\pi v^2} \frac{\cos\theta_T + \cos\theta_R}{\sqrt{R_T R_R}}. \tag{11}$$

Time-domain data $g(t, x_T, x_R)$ is obtained by applying inverse Fourier transform to the spectrum $g(\omega, x_T, x_R)$:

$$g(t, x_T, x_R) = \frac{1}{2\pi} \int g(\omega, x_T, x_R) e^{i\omega t} d\omega. \tag{12}$$

The only function in (9), which depends on ω, is s_{inv}. Using (11), integral with respect to ω can be calculated as

$$\frac{1}{2\pi} \int s_{inv}\, e^{i\omega t} d\omega = \hat{s}_{inv} \frac{\partial}{\partial t} \delta(t - t_{TR}), \tag{13}$$

where

$$t_{TR} = \frac{R_T + R_R}{v}. \tag{14}$$

Therefore, time-domain array data can be written as

$$g(t, x_T, x_R) = \frac{\partial}{\partial t} \int\int \left\{ \int \hat{s}_{inv}\, \delta(t - t_{TR})\, b(z, x'_T, x'_R) dz \right\} dx'_T dx'_R. \tag{15}$$

Integral with respect to z is calculated as

$$\{...\} = \hat{s}_{inv} b(z, x'_T, x'_R) \left| \frac{\partial(t - t_{TR})}{\partial z} \right|^{-1} \Bigg|_{z=z(t)}, \tag{16}$$

where $z(t)$ is solution to equation (14):

$$z(t) = \frac{\sqrt{\left(t^2 v^2 - r^2_{x(T)} - r^2_{x(R)}\right)^2 - 4 r^2_{x(T)} r^2_{x(R)}}}{2tv}, \quad r_{x(T,R)} = x_{T,R} - x'_{T,R}. \tag{17}$$

Finally,

$$g(t, x_T, x_R) = \frac{1}{2\pi v} \int\int \frac{1}{\sqrt{R_T R_R}} \frac{\partial}{\partial t} b\left(z(t), x'_T, x'_R\right) dx'_T dx'_R. \tag{18}$$

III. Data calibration and normalisation

All measured array datasets need to be calibrated. Moreover, the image amplitude is not spatially uniform and decreases with the imaging depth, mainly because of ultrasonic wave attenuation (which includes scattering induced attenuation and absorption). Therefore, in order to include data from neighbouring points into the local reference database, the image also has to be corrected. In this section the data calibration and image correction procedure is described.

Firstly, an average value of the root mean square (RMS) of image amplitude as a function of imaging depth is estimated using all reference images $I_n(x, z)$, $n = 1, ...N$:

$$I_{avr,RMS}(z) = \frac{1}{N} \sum_{n=1}^{N} \frac{I_{n,RMS}(z)}{I_{n,RMS}(z_{ref})}, \tag{19}$$

$$I_{n,RMS}(z) = \left(\frac{1}{L_x \Delta z} \int_{-L_x/2}^{L_x/2} \int_{z-\Delta z/2}^{z+\Delta z/2} |I_n(x, z)|^2 dz dx \right)^{1/2}, \tag{20}$$

where L_x is the image aperture, $\Delta z = 2\lambda$ and z_{ref} is an arbitrary defined reference depth. Functions $I_{avr,RMS}(z)$ for the numerical and two experimental examples are shown in Fig.2.

Then the image and the local array data are normalised as

$$I_c(x, z) = \frac{I(x, z)}{c_{norm}(z)}, \quad g_{c,local}(t, x_T, x_R | x, z) = \frac{g_{local}(t, x_T, x_R | x, z)}{c_{norm}(z)}, \tag{21}$$

where $c_{norm} = I_{RMS}(z_{ref}) I_{avr,RMS}(z)$ is the correction coefficient.

IV. Far field scattering amplitude

It is assumed that the region of interest with the center point (x, z) is located in the far-field from each array element. The schematic diagram is shown in Fig.1b. Then the frequency spectrum of the extracted and normalised

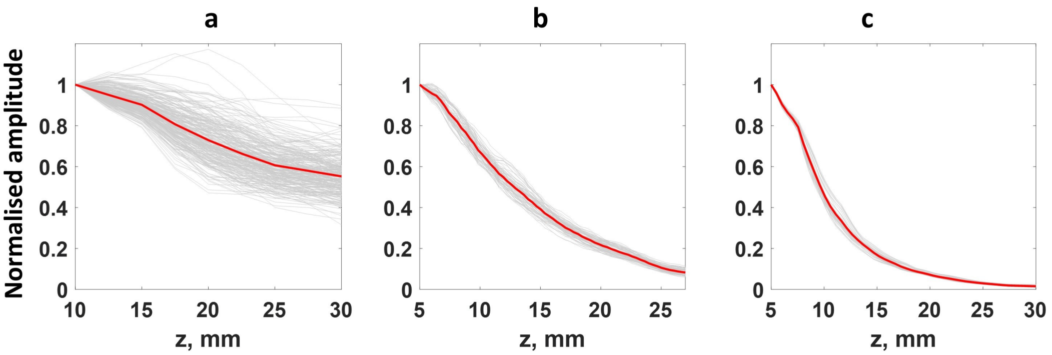

Fig. 2. **The root mean square of image amplitude as a function of imaging depth.** Gray lines correspond to individual reference images, the red line represents an average over all reference data. (a) Finite element model, 1 MHz. (b) Silicone-rubber specimen, 1.5 MHz. (c) Urethane-rubber specimen, 1.5 MHz.

local array data can be approximately written as:

$$g_{c,local}(\omega, x_T, x_R|x, z) = U_0 S(\theta_T, \theta_R, \omega)\, P_T P_R, \tag{22}$$

here U_0 is the normalisation constant, which is independent on the overall gain of the system. The factors $P_{T,R}$ describe the propagation of cylindrical wave from array element to the scatterer location,

$$P_{T,R} = D(\theta_{T,R})\sqrt{\frac{\lambda}{R_{T,R}}} e^{-ikR_{T,R}}, \tag{23}$$

where $R_{T,R} = \sqrt{(x - x_{T,R})^2 + z^2}$ and $D(\theta)$ is the directivity function of array element.

The complex valued function $S(\theta_T, \theta_R, \omega)$ is the far-field scattering amplitude or the scattering matrix of the scatterer. Conceptually, the scattering matrix provides the magnitude and phase of scattered waves in the far-field of the scatterer as a function of scattering angle, θ_R, for unit amplitude plane wave incident on the scatterer from angle θ_T (see Fig.1b).

The constant U_0 is the same for all measurement datasets, so the quantity $\hat{S} = U_0 S$ was extracted from the local array data as

$$\hat{S} = \frac{g_{c,local}}{P_T P_R}. \tag{24}$$

V. Multiple scattering rate

The multiple scattering rate is defined as a proportion of the multiple scattering contribution in the total image intensity. This quantity can be estimated directly from the generalised image [3]. The method is based on the specific structure of the single and multiple scattering components in the generalised image domain. The single scattering contribution is concentrated along the main diagonal of the generalised image, $x_T = x_R$. The multiple scattering contribution is spread over the whole generalised image domain, and the average multiple scattering intensity off

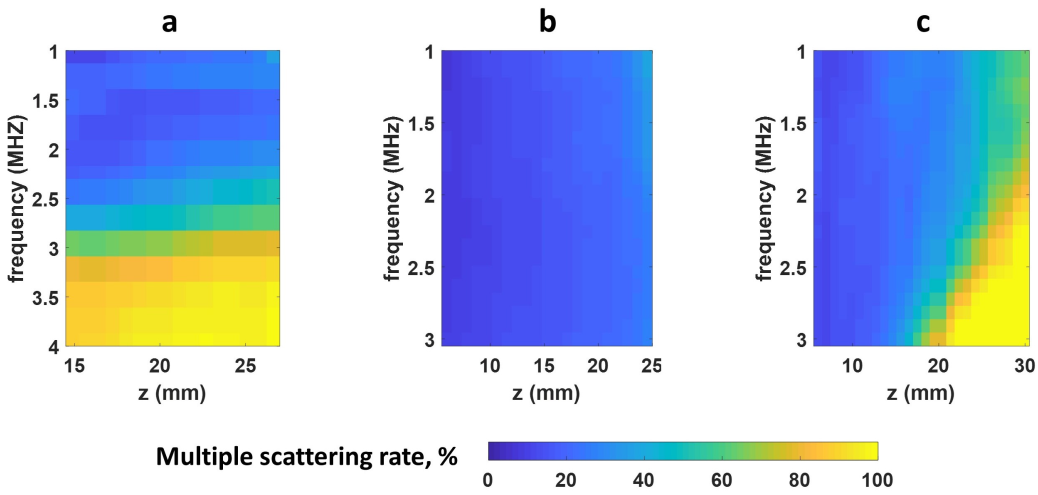

Fig. 3. **Multiple scattering rate in the image as a function of frequency and depth.** (a) Finite element model. (b) Silicone-rubber specimen. (c) Urethane-rubber specimen.

the main diagonal is a half of the average multiple scattering intensity on the main diagonal of the generalised image (the coherent backscattering effect).

Fig.3 shows the multiple scattering rate as a function of depth and frequency for the numerical and two experimental examples considered in the paper. The imaging frequency is a crucial parameter and its choice is determined by the requirement of predominantly single scattering regime. For the considered examples we estimated, that the multiple scattering rate has to be approximately smaller than 25% in order to achieve sufficient signal to noise ratio for the local scattering imaging. Note that for the experimental samples another limiting factor affecting the imaging frequency was array element under-sampling and high absorption at higher frequencies.

VI. Global probability of false alarm

The global false alarm rate represents a probability that at least one pixel on the image, $I(x,z)$, exceeds a given threshold t, $P(I(x,z)\geq t)$, and corresponds to the global p-value. For the Gaussian random field and the rectangular imaging area $L_x \times L_z$, an analytical expression was derived by Worsley $et\ al.$ [4]. The size of the area is defined in terms of resolution elements, or resels, as

$$\hat{L}_x = \frac{L_x}{d_{x,res}}, \quad \hat{L}_z = \frac{L_z}{d_{z,res}}, \tag{25}$$

where $d_{x,res}$ and $d_{z,res}$ is the resel size in x and z directions, respectively. Then $P(I\geq t)$ is given by

$$P(I\geq t) = \rho_0(t) + (\hat{L}_x + \hat{L}_z)\rho_1(t) + \hat{L}_x\hat{L}_z\rho_2(t), \tag{26}$$

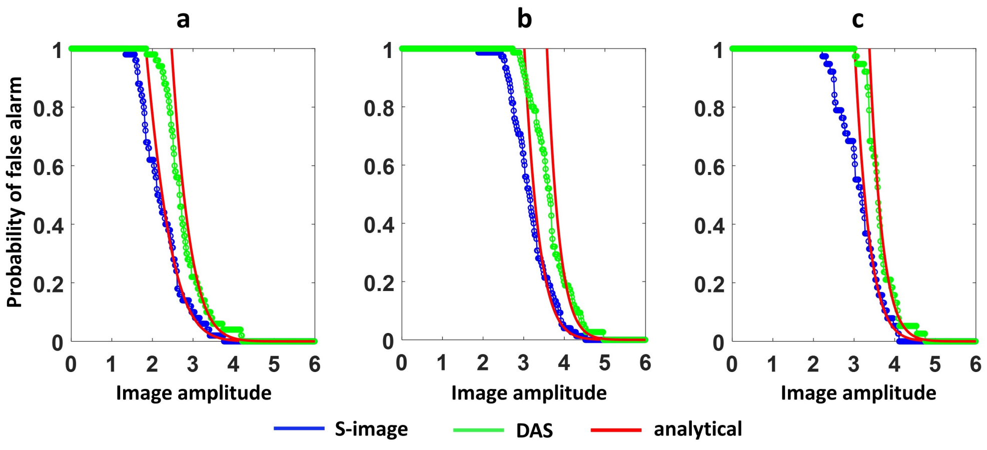

Fig. 4. **Global probability of false alarm.** (a) Finite element model, 1 MHz. (b) Silicone-rubber specimen, fused images at 1.2, 1.5, 1.8 MHz. (c) Urethane-rubber specimen, fused images at 1.2, 1.5, 2 MHz. Blue, green and red curves correspond to local scattering images, delay-and-sum images and analytical expression (26), respectively.

where

$$\rho_0(t)=\frac{1}{\sqrt{2\pi}}\int_t^{\infty}e^{-u^2/2}du=\frac{1}{2}\left(1-\mathrm{erf}\left(\frac{t}{\sqrt{2}}\right)\right),\quad \rho_1(t)=\frac{\sqrt{4\ln 2}}{2\pi}e^{-t^2/2},\quad \rho_2(t)=\frac{4\ln 2}{(2\pi)^{3/2}}e^{-t^2/2}t, \tag{27}$$

here erf(t) is the error function. Note that expression (26) provides a good approximation for high thresholds (and low probabilities of false alarm). For simplicity it is assumed, that each resel represents a square with the size d_{res}, so $d_{x,res}=d_{z,res}=d_{res}$. The function (26) was parametrically fitted to the experimentally estimated probability of false alarm (based on the reference data) and results are shown in Fig.4 for the numerical and two experimental examples. The corresponding resel sizes are given in Table I. It can be seen, that, in general, a good fit is achieved for the false alarm rate $P\leq 0.3$.

TABLE I
RESEL SIZE.

	resel size, mm	
	DAS	S-image
Finite element model, 1 MHz	3.7 (0.8λ at 1MHz)	7.2 (1.5λ at 1MHz)
Silicone-rubber sample, fused images at 1.2, 1.5, 1.8 MHz	0.8 (1.2λ at 1.5MHz)	1.9 (2.8λ at 1.5MHz)
Urethane-rubber sample, fused images at 1.2, 1.5, 2 MHz	1.1 (1.1λ at 1.5MHz)	1.9 (1.9λ at 1.5MHz)

VII. Statistical results for the finite element model

Detailed description of the Finite Element modelling procedure can be found in [5]. The specimen was 40 mm deep and the material properties were chosen close to the copper as $c_{11}=168.6$GPa, $c_{12}=121.4$GPa, $c_{44}=75.4$GPa and $\rho=8960$kg/m^3 (c_{11}, c_{12}, and c_{44} are elastic constants of a cubic material). The longitudinal velocity in this case is 4690 m/s.

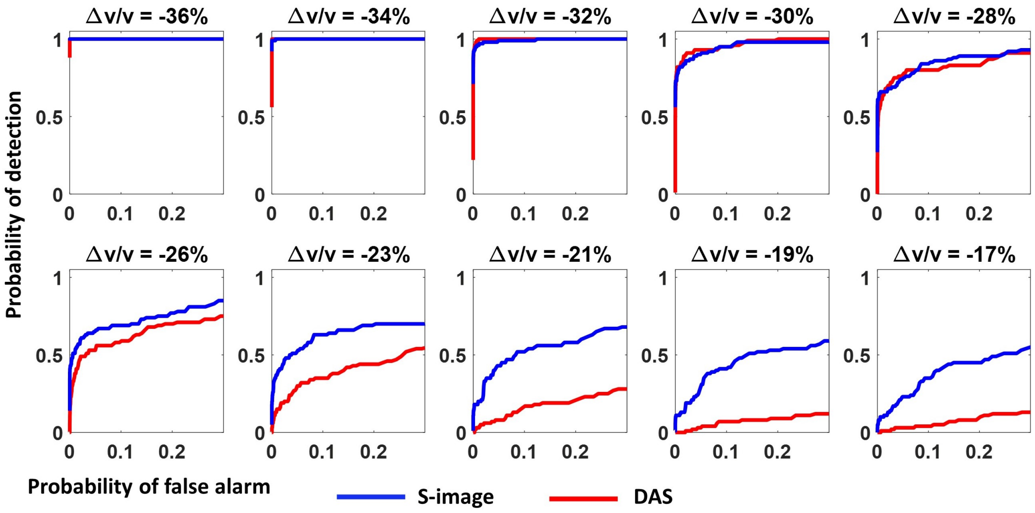

Fig. 5. Receiver operating characteristic (ROC) curves for the FE model of inclusions with different longitudinal velocity contrast $\Delta v/v$.

In order to study the statistical performance of the local scattering imaging method, 10 different inclusion types with various sound speeds and 100 different realisations of grain structure for each inclusion type were modelled. Each inclusion was 2 mm diameter and located at 20 mm depth directly below the array. Detection rate was evaluated using receiver operating characteristic (ROC) curve. Each inclusion was considered as detected if the image amplitude was above the threshold in the area 5 mm $\times$ 5 mm around the center of the inclusion. The results, presented in Fig.5, show that the detection rate based on the delay-and-sum image drops very fast when the contrast of inclusion decreases, while the probability of detection based on the local scattering image decreases much more slowly.

The performance of the delay-and-sum and the local scattering methods can also be assessed by evaluating the probability of detection as a function of the noise level. It was mentioned that the local scattering image amplitude (z-score) can also be interpreted as the Contrast-to-Noise ratio (CNR). In this case the Contrast-to-Noise ratio is defined as a ratio of the difference between the signal and average noise to the standard deviation of the noise. For the standard normal distribution (z-score) the mean is zero and the standard deviation is one, so the Contrast-to-Noise ratio in this case equals the image amplitude. Fig.6 shows the probability of detection as a function of the mean CNR level, corresponding to different inclusion types. The probability of false alarms was set equal to 1%. It is seen that as the CNR decreases the performance of the delay-and-sum method rapidly degrades, while the local scattering approach exhibits a more gradual decline and significantly outperforms the delay-and-sum method at low CNR values. For example, for CNR levels equal to 3 the delay-and-sum method detects only 8% of inclusions, while the local scattering method increases the detection rate to 40%.

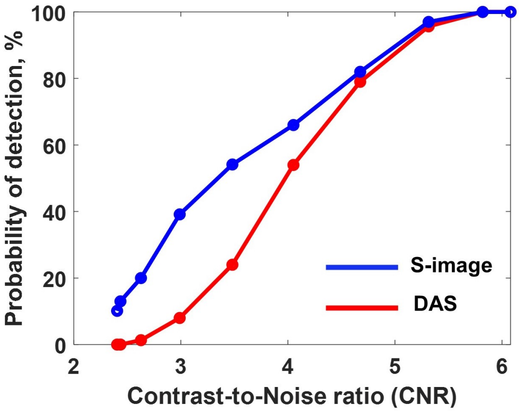

Fig. 6. Probability of detection for the FE model of different inclusions as a function of the mean Contrast-to-Noise ratio (CNR). The probability of false alarms is 1%.

References

[1] Velichko, A., Wilcox, P. D. Reversible back-propagation imaging algorithm for post-processing of ultrasonic array data. *IEEE Trans. Ultrason. Ferroelectr. Freq. Control* vol.56, no.11, 2492-2503 (2009).

[2] Velichko, A., Wilcox, P. D. An analytical comparison of ultrasonic array imaging algorithms. *J. Acoust. Soc. Am.*, vol. 127, no. 4, 2377-2384 (2010).

[3] Velichko, A. Quantification of the effect of multiple scattering on array imaging performance. *IEEE Trans. Ultrason. Ferroelectr. Freq. Control*, vol.67, no.1, 92-105 (2020).

[4] Worsley, K. J. et al. A Unified Statistical Approach for Determining Significant Signals in Images of Cerebral Activation. *Human Brain Mapping* **4**, 458-73 (1996).

[5] Bloxham, H. A., Velichko, A., and Wilcox, P. D. Establishing the limits of validity of the superposition of experimental and analytical ultrasonic responses for simulating imaging data. *IEEE Trans. Ultrason. Ferroelectr. Freq. Control* vol. 66, 101-108 (2019).

.
